# Annotations

**Published:** 1902-12-20

**Authors:** 


### Annotations.

### The London Ambulance Question.

The problem of providing an efficient and complete ambulance service for the metropolis is a very complex one, the difficulties connected with it arising partly from the enormous size of London, partly from the concentration of the hospitals in the central area. A time may come when London may find itself provided with ambulances on the plan which prevails in New York, where, in connection with the hospitals, a system of horse-ambulances is established almost as complete as that of the fire brigade, a system under which on the receipt of a "call" a horse ambulance is at once dispatched carrying with it a medical officer and a proper supply of surgical appliances and emergency medicines, or on the plan which obtains in Liverpool, where, although most of the ambulances are kept at the hospitals the whole system is worked by the police in connection with their telephone and signalling system. In the meanwhile, however, in dealing with an area so large as London, it is obvious that very special arrangements will have to be made. At the present time the police possess a certain number of wheeled hand litters, and the Bischoffsheim Ambulance Association has a large number of the well-known street ambulances scattered about in prominent positions, while the St. John Ambulance Association has ambulances at its stations, and horsed ambulances can be obtained from its central office. But no means exist of linking up the different parts of the system. What is in our opinion wanted, short of a complete system on the New York plan which we fear is too great a thing to hope for, is on the one hand a considerable extension of the street ambulance system as it now exists—spreading it widely into the outlying districts where, from the absence of cabs (a ready although defective substitute), the want of ambulances is a really very serious matter—an improvement of the pattern of the police stretcher, and a complete system of horse ambulances, to connect the outlying with the central districts. If one had to build London afresh, one would without doubt spread the hospitals out much more than at present is the case, and that also may come. But so long as the hospitals are centrally placed, the first need of the outlying districts is easy ambulance communication with the nearest, although still far distant, hospital. More street ambulances, a definite arrangement for rewarding the police for the extra work involved in removing accidents to hospitals, and a liberal supply of horse ambulances in the outlying districts would go far to relieve the present anomalous condition of affairs.

### The Children of Alcoholic Parents.

That a good many alcoholic parents have children who themselves become alcoholic can hardly be denied. Whether they suffer because of their parents' fault is another matter; probably when heredity has anything to do with it parent and offspring alike are the victims of some old taint of nervous instability which has come down through many generations. But however it happens, we cannot, in the bringing up of a child, ignore its tendencies as shown by those of its progenitors. Hence it becomes a very important question to decide, from quite early days, what sort of a régime such a child should be subjected to. Such children are often liable to exhibit extremes of activity and prostration. In work, in study, and equally in play, they show enormous energy and "go" for a short time, and this is followed by periods of exhaustion. As these children grow up they are neurotic, often brilliant in certain ways, but very impatient of suffering, and it is to relieve their phases of depression that later on alcohol is so often taken. In the rearing of such children then nothing should be done to increase their inherent tendency to sudden outbursts of energy. Stimulants should be absolutely avoided, and one must not confine the term to alcoholic stimulants alone. Our object should be to produce a slow and passive growth rather than a sudden evolution of nerve force, hence alcohol, tea, coffee, and even meat broths should be forbidden. In the daily diet also meat should be given in very limited quantities, or is perhaps better avoided altegether, the diet being made to consist of milk and vegetables, fruit and farinaceous foods. The meals should be administered at regular times, and under no circumstances should such children be allowed to relieve any sense of emptiness or faintness by intermediate snacks. This habit of immediately relieving every internal discomfort by putting something into the stomach is at the root of the evil. In childhood it leads to biscuits and sweets, but in manhood it too often leads to unlimited "nips" and "pegs" and other easy means of relieving depression. The origin of tippling may sometimes be traced back to nursery habits.

### The Barber's Chair.

In the early days of the existence of The Hospital attention was called in its columns to the danger of contracting skin diseases that arise from the carelessness which barbers so frequently show in regard to their clients. We are therefore gratified to see that the Board of Public Health of the city of Toronto has issued regulations for barbers with regard to patients suffering from contagious skin diseases, and that the Hairdressers' Association of the same place has agreed to accept these. It cannot be said that they are all that is required, but they are a step in the right direction. Customers suffering from any such diseases are to be attended at their own homes, and the razors and scissors used for them are always to be sterilised. That is good so far as it goes, but who is a barber—now that he has parted company with the surgeon—that he should recognise when a customer is or is not suffering from a contagious skin disease? Would it not be safer as well as simpler and less offensive to the susceptibilities of the client, to treat every customer as a potential sufferer? Let the razors, the scissors, and the comb be sterilised after each operation. If a brush is used, let it be newly washed, and let a clean antimacassar be placed on the back of the chair, before a new customer is asked to lay his head upon it. Even if these improvements cost a little money, for the purchase of extra utensils, we think the man who dared to start this innovation would reap his reward when people realised the risks from which he saved them, and before long public opinion would bring all respectable barbers up to the same standard of cleanliness.

